# 3-(2-Chloro­phen­yl)-4-(4-nitro­phen­yl)-1*H*-1,2,4-triazole-5(4*H*)-thione

**DOI:** 10.1107/S1600536811020459

**Published:** 2011-06-11

**Authors:** Abbas Nikoo, Karim Akbari Dilmaghani, Ali Hassanzadeh, Behrouz Notash

**Affiliations:** a Department of Chemistry, Faculty of Science, Urmia University, 57159 Urmia, Iran; b Department of Chemistry, Shahid Beheshti University, G. C. Evin, Tehran 1983963113, Iran

## Abstract

In the crystal structure of the title triazole compound, C_14_H_9_ClN_4_O_2_S, mol­ecules are connected into centrosymmetric dimers by pairs of N—H⋯S hydrogen bonds. In addition, there are weak C—H⋯N hydrogen bonds stabilizing the crystal structure. The dihedral angles between the triazole ring and the two benzene rings are 73.0 (4) and 72.9 (4)°.

## Related literature

For related structures, see: Genç *et al.* (2004 ▶); Kumaran *et al.* (1999 ▶). For the synthesis of triazoles, see: Zamani *et al.* (2003 ▶).
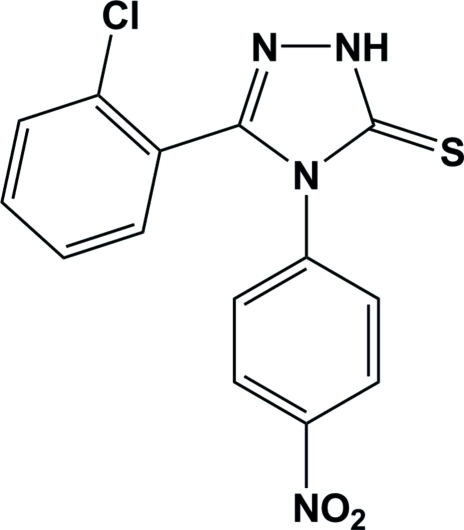


## Experimental

### 

#### Crystal data

C_14_H_9_ClN_4_O_2_S
*M* *_r_* = 332.77
Monoclinic, 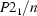

*a* = 6.7262 (13) Å
*b* = 17.109 (3) Å
*c* = 13.101 (3) Å
β = 95.89 (3)°
*V* = 1499.7 (5) Å^3^
*Z* = 4
Mo *K*α radiation
μ = 0.41 mm^−1^
*T* = 298 K
0.35 × 0.3 × 0.3 mm

#### Data collection

Stoe IPDS 2T diffractometer
16462 measured reflections
4038 independent reflections
2850 reflections with *I* > 2σ(*I*)
*R*_int_ = 0.060

#### Refinement

*R*[*F* ^2^ > 2σ(*F* ^2^)] = 0.072
*wR*(*F* ^2^) = 0.174
*S* = 1.18
4038 reflections
203 parameters
H atoms treated by a mixture of independent and constrained refinement
Δρ_max_ = 0.26 e Å^−3^
Δρ_min_ = −0.23 e Å^−3^

### 

Data collection: *X-AREA* (Stoe & Cie, 2005 ▶); cell refinement: *X-AREA*; data reduction: *X-AREA*; program(s) used to solve structure: *SHELXS97* (Sheldrick, 2008 ▶); program(s) used to refine structure: *SHELXL97* (Sheldrick, 2008 ▶); molecular graphics: *ORTEP-3 for Windows* (Farrugia, 1997 ▶); software used to prepare material for publication: *WinGX* (Farrugia, 1999 ▶).

## Supplementary Material

Crystal structure: contains datablock(s) I, global. DOI: 10.1107/S1600536811020459/bt5548sup1.cif

Structure factors: contains datablock(s) I. DOI: 10.1107/S1600536811020459/bt5548Isup2.hkl

Supplementary material file. DOI: 10.1107/S1600536811020459/bt5548Isup3.cml

Additional supplementary materials:  crystallographic information; 3D view; checkCIF report

## Figures and Tables

**Table 1:** Hydrogen-bond geometry (Å, °)

*D*—H⋯*A*	*D*—H	H⋯*A*	*D*⋯*A*	*D*—H⋯*A*
N2—H1⋯S1^i^	0.86 (3)	2.48 (3)	3.328 (3)	172 (3)
C2—H2⋯N1^ii^	0.93	2.54	3.454 (5)	170

Symmetry codes: (i) 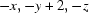; (ii) 
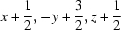
.

**Fig. 1.**
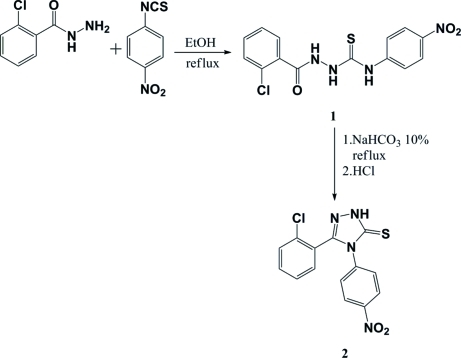
The reaction scheme for synthesis of the title compound.

**Fig. 2.**
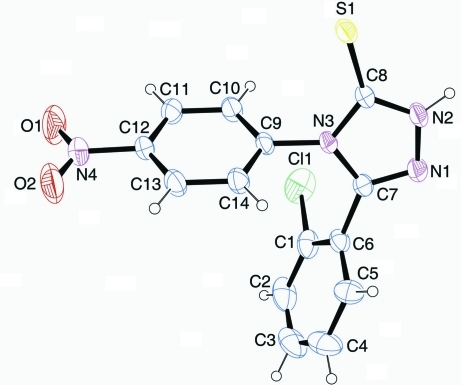
The molecular structure of the title compound with displacement ellipsoids drawn at 30% probability level.

**Fig. 3.**
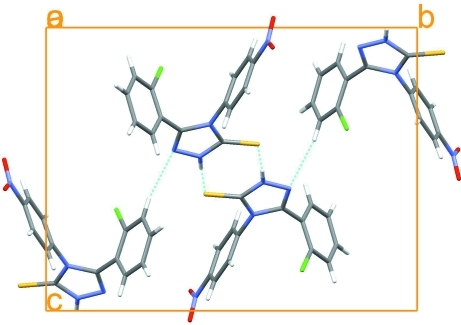
The packing diagram of the title compound down the a axis. The intermolecular N—H···S, C—H···N hydrogen bonds are shown as blue dashed lines.

## supplementary crystallographic information

### Comment

In the medicinal chemistry, 1,2,4-triazoles are widely used. Cyclization of 1,4-disubstituted thiosemicarbazides produced 4,5-disubstituted 1,2,4-triazoles (Zamani *et al.*, 2003). 4-nitro phenylisothiocyanate reacted with 2-chlorophenylcarboxylic acid hydrazide to yield the corresponding 1-(2-chlorobenzoyl)-4-(4-nitrophenyl)thiosemicarbazide (1), whereas cyclization of (1) with NaHCO_3_ 10% solution gave the 3-(2-Chlorophenyl)-4-(4-nitrophenyl)-1*H*-1,2,4-triazole-5(4*H*)-thione (2) (Fig. 1).The structures of the compounds were assigned on the basis of IR, ^1^H-NMR and ^13^C-NMR spectra.

The molecular structure of the title compound is shown in Fig. 2. In the crystal structure of the title compound, there are intermolecular N—H···S and weak C—H···N hydrogen bonding which play important role in the stabilization of the crystal structure (Table 1 and Fig. 3).

### Experimental

Starting materials were obtained from Merck. For the synthesis of 1-(2-chlorobenzoyl)-4-(4-nitrophenyl)thiosemicarbazide (1), a mixture of 2-chlorophenylcarboxylic acid hydrazide (0.01 mol, 1.7 g) and 4-nitrophenyl isothiocynate (0.01 mol, 1.8 g) in absolute ethanol was refluxed for 6 h. The solid material obtained on cooling was filtered, washed with diethyl ether, dried and crystallized from ethanol (yield 82%; m.p. 170–172°C). IR (KBr, cm^-1^): 3315, 3184 (N—H), 1643 (C═O), 1457, 1330 (NO_2_), 1273 (C═S); ^1^H NMR (300 MHz, DMSO-d_6_): 7.42–7.53 (3*H*, m, 2-chlorophenyl), 7.74 (1*H*, s, 2-chlorophenyl), 7.90 (2*H*, d, J = 8.7, Ar—H), 8.21 (2*H*, d, J = 8.7, Ar—H), 9.99 (1*H*, br, –NH—Ar), 10.30 (1*H*, s, –CS—NH–), 10.56 (1*H*, br, –CO—NH–); ^13^C NMR (75 MHz, DMSO-d_6_): 121.59, 124.66, 125.11, 127.43, 130.39, 131.22, 132.19, 146.25, 165.93 and 181.58. For the synthesis of (2), a stirred mixture of (1) (1 mmol, 0.35 g) and NaHCO_3_ 10% (10 ml) was refluxed for 6 h. After cooling, the solution was acidified with hydrochloric acid and the precipitate was filtered. The precipitate was then crystallized from ethanol (yield 57%; m.p. 223–225°C). IR (KBr, cm^-1^): 3286 (N—H), 1608 (C═N), 1465, 1336 (NO_2_), 1529, 1177, 1071, 963 (N—═S, amide I, II, III and IV bands); ^1^H NMR (300 MHz, CDCl_3_): 7.37–7.53 (6*H*, m, Ar—H), 7.91 (1*H*, s, 2-chlorophenyl), 8.23 (2*H*, d, J = 8.7, Ar—H), 12.24 (1*H*, s, SH); ^13^C NMR (75 MHz, CDCl_3_): 124.43, 127.26, 127.48, 128.74, 130.30, 130.50, 131.58, 132.23, 133, 133.07.

### Refinement

The H atom attached to amine group was found in a difference Fourier map and refined isotropically without restraint. The C—H protons were positioned geometrically and refined as riding atoms with C—H = 0.93 Å and *U*iso(H) = 1.2 *U*eq(C).

### Crystal data

**Table:**

C_14_H_9_ClN_4_O_2_S	*F*(000) = 680.0
*M**_r_* = 332.77	*D*_x_ = 1.474 Mg m^−^^3^
Monoclinic, *P*2_1_/*n*	Mo *K*α radiation, λ = 0.71073 Å
Hall symbol: -P 2yn	Cell parameters from 4038 reflections
*a* = 6.7262 (13) Å	θ = 2.4–29.2°
*b* = 17.109 (3) Å	µ = 0.41 mm^−^^1^
*c* = 13.101 (3) Å	*T* = 298 K
β = 95.89 (3)°	Block, brown
*V* = 1499.7 (5) Å^3^	0.35 × 0.3 × 0.3 mm
*Z* = 4	

### Data collection

**Table:**

Stoe IPDS 2T diffractometer	2850 reflections with *I* > 2σ(*I*)
Radiation source: fine-focus sealed tube	*R*_int_ = 0.060
graphite	θ_max_ = 29.2°, θ_min_ = 2.4°
Detector resolution: 0.15 pixels mm^-1^	*h* = −9→9
rotation method scans	*k* = −23→22
16462 measured reflections	*l* = −16→17
4038 independent reflections	

### Refinement

**Table:**

Refinement on *F*^2^	Primary atom site location: structure-invariant direct methods
Least-squares matrix: full	Secondary atom site location: difference Fourier map
*R*[*F*^2^ > 2σ(*F*^2^)] = 0.072	Hydrogen site location: inferred from neighbouring sites
*wR*(*F*^2^) = 0.174	H atoms treated by a mixture of independent and constrained refinement
*S* = 1.18	*w* = 1/[σ^2^(*F*_o_^2^) + (0.0706*P*)^2^ + 0.4737*P*] where *P* = (*F*_o_^2^ + 2*F*_c_^2^)/3
4038 reflections	(Δ/σ)_max_ = 0.001
203 parameters	Δρ_max_ = 0.26 e Å^−^^3^
0 restraints	Δρ_min_ = −0.23 e Å^−^^3^

### Special details

**Table:**

Geometry. All e.s.d.'s (except the e.s.d. in the dihedral angle between two l.s. planes) are estimated using the full covariance matrix. The cell e.s.d.'s are taken into account individually in the estimation of e.s.d.'s in distances, angles and torsion angles; correlations between e.s.d.'s in cell parameters are only used when they are defined by crystal symmetry. An approximate (isotropic) treatment of cell e.s.d.'s is used for estimating e.s.d.'s involving l.s. planes.
Refinement. Refinement of *F*^2^ against ALL reflections. The weighted *R*-factor *wR* and goodness of fit *S* are based on *F*^2^, conventional *R*-factors *R* are based on *F*, with *F* set to zero for negative *F*^2^. The threshold expression of *F*^2^ > σ(*F*^2^) is used only for calculating *R*-factors(gt) *etc*. and is not relevant to the choice of reflections for refinement. *R*-factors based on *F*^2^ are statistically about twice as large as those based on *F*, and *R*-factors based on ALL data will be even larger.

### Fractional atomic coordinates and isotropic or equivalent isotropic displacement parameters (Å^2^)

**Table:**

	*x*	*y*	*z*	*U*_iso_*/*U*_eq_	
S1	0.23357 (11)	1.07003 (4)	0.08833 (6)	0.0535 (2)	
Cl1	0.4224 (2)	0.80997 (7)	0.35611 (8)	0.0955 (4)	
N3	0.4499 (3)	0.94203 (12)	0.15936 (15)	0.0400 (5)	
C8	0.2956 (4)	0.97505 (15)	0.09792 (19)	0.0417 (6)	
C7	0.4517 (4)	0.86289 (16)	0.13680 (19)	0.0433 (6)	
C9	0.5843 (4)	0.98312 (15)	0.23334 (19)	0.0403 (5)	
N2	0.2132 (4)	0.91476 (14)	0.04511 (19)	0.0512 (6)	
C14	0.7779 (4)	0.99626 (19)	0.2123 (2)	0.0538 (7)	
H14	0.8207	0.9795	0.1506	0.065*	
C12	0.8404 (4)	1.05784 (17)	0.3744 (2)	0.0512 (7)	
N1	0.3080 (4)	0.84485 (14)	0.06799 (19)	0.0523 (6)	
C6	0.6088 (4)	0.80956 (16)	0.1831 (2)	0.0479 (6)	
C11	0.6475 (5)	1.04638 (19)	0.3956 (2)	0.0555 (7)	
H11	0.6049	1.0640	0.4569	0.067*	
C5	0.7588 (5)	0.78584 (19)	0.1248 (3)	0.0644 (9)	
H5	0.7544	0.8011	0.0565	0.077*	
N4	0.9821 (5)	1.09731 (19)	0.4514 (2)	0.0732 (8)	
C1	0.6151 (5)	0.78478 (19)	0.2838 (2)	0.0612 (8)	
C13	0.9084 (4)	1.0349 (2)	0.2844 (2)	0.0598 (8)	
H13	1.0395	1.0449	0.2716	0.072*	
C10	0.5174 (4)	1.00804 (19)	0.3240 (2)	0.0508 (7)	
H10	0.3858	0.9991	0.3367	0.061*	
O2	1.1498 (5)	1.1103 (2)	0.4306 (3)	0.1210 (13)	
C2	0.7732 (8)	0.7390 (2)	0.3270 (3)	0.0859 (13)	
H2	0.7789	0.7230	0.3951	0.103*	
O1	0.9257 (5)	1.1125 (2)	0.5332 (2)	0.1153 (12)	
C3	0.9209 (7)	0.7181 (2)	0.2669 (5)	0.0946 (15)	
H3	1.0281	0.6883	0.2954	0.114*	
C4	0.9137 (6)	0.7400 (2)	0.1668 (4)	0.0852 (13)	
H4	1.0131	0.7240	0.1270	0.102*	
H1	0.104 (5)	0.9176 (17)	0.006 (2)	0.048 (8)*	

### Atomic displacement parameters (Å^2^)

**Table:**

	*U*^11^	*U*^22^	*U*^33^	*U*^12^	*U*^13^	*U*^23^
S1	0.0494 (4)	0.0469 (4)	0.0592 (4)	0.0006 (3)	−0.0187 (3)	0.0028 (3)
Cl1	0.1317 (10)	0.1029 (8)	0.0554 (5)	−0.0121 (7)	0.0263 (6)	0.0092 (5)
N3	0.0405 (11)	0.0458 (12)	0.0315 (10)	0.0015 (9)	−0.0071 (8)	0.0005 (8)
C8	0.0380 (12)	0.0494 (14)	0.0360 (12)	−0.0011 (10)	−0.0041 (10)	0.0027 (10)
C7	0.0465 (14)	0.0479 (14)	0.0341 (12)	0.0028 (11)	−0.0024 (10)	0.0026 (10)
C9	0.0384 (12)	0.0472 (14)	0.0328 (12)	0.0008 (10)	−0.0081 (10)	0.0017 (10)
N2	0.0500 (13)	0.0513 (14)	0.0476 (13)	0.0014 (10)	−0.0187 (11)	−0.0010 (10)
C14	0.0428 (15)	0.077 (2)	0.0415 (15)	0.0000 (13)	0.0016 (12)	−0.0100 (13)
C12	0.0479 (15)	0.0567 (17)	0.0453 (15)	0.0043 (12)	−0.0134 (12)	−0.0085 (12)
N1	0.0579 (14)	0.0506 (13)	0.0450 (13)	0.0029 (11)	−0.0112 (11)	−0.0022 (10)
C6	0.0503 (15)	0.0420 (14)	0.0485 (15)	−0.0010 (11)	−0.0094 (12)	0.0050 (11)
C11	0.0558 (17)	0.0692 (19)	0.0408 (15)	0.0009 (14)	0.0015 (13)	−0.0119 (13)
C5	0.065 (2)	0.0530 (18)	0.075 (2)	0.0103 (15)	0.0080 (17)	0.0146 (15)
N4	0.0633 (18)	0.085 (2)	0.0659 (19)	0.0037 (15)	−0.0195 (15)	−0.0246 (15)
C1	0.082 (2)	0.0535 (17)	0.0446 (16)	−0.0095 (15)	−0.0103 (15)	0.0079 (13)
C13	0.0355 (14)	0.084 (2)	0.0592 (18)	−0.0052 (14)	−0.0003 (12)	−0.0125 (16)
C10	0.0401 (14)	0.0705 (19)	0.0415 (14)	−0.0031 (12)	0.0038 (11)	−0.0056 (13)
O2	0.0646 (18)	0.172 (3)	0.122 (3)	−0.030 (2)	−0.0108 (17)	−0.065 (2)
C2	0.116 (3)	0.063 (2)	0.069 (2)	−0.007 (2)	−0.039 (2)	0.0228 (18)
O1	0.100 (2)	0.168 (3)	0.074 (2)	−0.015 (2)	−0.0115 (17)	−0.061 (2)
C3	0.082 (3)	0.057 (2)	0.134 (4)	0.0075 (19)	−0.043 (3)	0.018 (2)
C4	0.064 (2)	0.056 (2)	0.135 (4)	0.0139 (17)	0.008 (2)	0.018 (2)

### Geometric parameters (Å, °)

**Table:**

S1—C8	1.679 (3)	C6—C1	1.382 (4)
Cl1—C1	1.736 (4)	C6—C5	1.387 (5)
N3—C8	1.369 (3)	C11—C10	1.382 (4)
N3—C7	1.386 (3)	C11—H11	0.9300
N3—C9	1.439 (3)	C5—C4	1.373 (5)
C8—N2	1.331 (3)	C5—H5	0.9300
C7—N1	1.290 (3)	N4—O1	1.203 (4)
C7—C6	1.479 (4)	N4—O2	1.208 (4)
C9—C14	1.377 (4)	C1—C2	1.393 (5)
C9—C10	1.380 (4)	C13—H13	0.9300
N2—N1	1.374 (3)	C10—H10	0.9300
N2—H1	0.86 (3)	C2—C3	1.377 (7)
C14—C13	1.389 (4)	C2—H2	0.9300
C14—H14	0.9300	C3—C4	1.360 (7)
C12—C13	1.366 (4)	C3—H3	0.9300
C12—C11	1.369 (4)	C4—H4	0.9300
C12—N4	1.478 (4)		
			
C8—N3—C7	107.5 (2)	C12—C11—H11	120.7
C8—N3—C9	125.5 (2)	C10—C11—H11	120.7
C7—N3—C9	127.0 (2)	C4—C5—C6	120.7 (4)
N2—C8—N3	103.6 (2)	C4—C5—H5	119.7
N2—C8—S1	128.6 (2)	C6—C5—H5	119.7
N3—C8—S1	127.68 (19)	O1—N4—O2	123.3 (3)
N1—C7—N3	111.1 (2)	O1—N4—C12	117.8 (3)
N1—C7—C6	126.3 (2)	O2—N4—C12	118.9 (3)
N3—C7—C6	122.4 (2)	C6—C1—C2	120.5 (4)
C14—C9—C10	121.4 (2)	C6—C1—Cl1	119.5 (3)
C14—C9—N3	119.1 (2)	C2—C1—Cl1	120.0 (3)
C10—C9—N3	119.5 (2)	C12—C13—C14	118.7 (3)
C8—N2—N1	113.7 (2)	C12—C13—H13	120.6
C8—N2—H1	124 (2)	C14—C13—H13	120.6
N1—N2—H1	122 (2)	C9—C10—C11	119.4 (3)
C9—C14—C13	119.1 (3)	C9—C10—H10	120.3
C9—C14—H14	120.5	C11—C10—H10	120.3
C13—C14—H14	120.5	C3—C2—C1	118.6 (4)
C13—C12—C11	122.8 (3)	C3—C2—H2	120.7
C13—C12—N4	118.1 (3)	C1—C2—H2	120.7
C11—C12—N4	119.1 (3)	C4—C3—C2	121.6 (4)
C7—N1—N2	104.0 (2)	C4—C3—H3	119.2
C1—C6—C5	118.9 (3)	C2—C3—H3	119.2
C1—C6—C7	122.1 (3)	C3—C4—C5	119.7 (4)
C5—C6—C7	118.9 (3)	C3—C4—H4	120.2
C12—C11—C10	118.6 (3)	C5—C4—H4	120.2
			
C7—N3—C8—N2	1.3 (3)	C13—C12—C11—C10	1.8 (5)
C9—N3—C8—N2	−178.7 (2)	N4—C12—C11—C10	−178.7 (3)
C7—N3—C8—S1	−176.1 (2)	C1—C6—C5—C4	−1.5 (5)
C9—N3—C8—S1	3.8 (4)	C7—C6—C5—C4	176.2 (3)
C8—N3—C7—N1	−1.3 (3)	C13—C12—N4—O1	−174.9 (4)
C9—N3—C7—N1	178.7 (3)	C11—C12—N4—O1	5.6 (5)
C8—N3—C7—C6	175.2 (2)	C13—C12—N4—O2	2.6 (5)
C9—N3—C7—C6	−4.8 (4)	C11—C12—N4—O2	−176.9 (4)
C8—N3—C9—C14	−107.0 (3)	C5—C6—C1—C2	2.2 (5)
C7—N3—C9—C14	72.9 (4)	C7—C6—C1—C2	−175.4 (3)
C8—N3—C9—C10	73.4 (3)	C5—C6—C1—Cl1	−176.9 (2)
C7—N3—C9—C10	−106.6 (3)	C7—C6—C1—Cl1	5.4 (4)
N3—C8—N2—N1	−1.0 (3)	C11—C12—C13—C14	−1.9 (5)
S1—C8—N2—N1	176.4 (2)	N4—C12—C13—C14	178.7 (3)
C10—C9—C14—C13	0.5 (5)	C9—C14—C13—C12	0.7 (5)
N3—C9—C14—C13	−179.0 (3)	C14—C9—C10—C11	−0.6 (5)
N3—C7—N1—N2	0.7 (3)	N3—C9—C10—C11	179.0 (3)
C6—C7—N1—N2	−175.6 (3)	C12—C11—C10—C9	−0.6 (5)
C8—N2—N1—C7	0.2 (3)	C6—C1—C2—C3	−1.0 (5)
N1—C7—C6—C1	−109.4 (4)	Cl1—C1—C2—C3	178.1 (3)
N3—C7—C6—C1	74.7 (4)	C1—C2—C3—C4	−1.0 (6)
N1—C7—C6—C5	73.0 (4)	C2—C3—C4—C5	1.8 (6)
N3—C7—C6—C5	−103.0 (3)	C6—C5—C4—C3	−0.5 (6)

### Hydrogen-bond geometry (Å, °)

**Table:**

*D*—H···*A*	*D*—H	H···*A*	*D*···*A*	*D*—H···*A*
N2—H1···S1^i^	0.86 (3)	2.48 (3)	3.328 (3)	172 (3)
C2—H2···N1^ii^	0.93	2.54	3.454 (5)	170.

Symmetry codes: (i) −*x*, −*y*+2, −*z*; (ii) *x*+1/2, −*y*+3/2, *z*+1/2.

## References

[bb1] Farrugia, L. J. (1997). *J. Appl. Cryst.* **30**, 565.

[bb2] Farrugia, L. J. (1999). *J. Appl. Cryst.* **32**, 837–838.

[bb3] Genç, S., Dege, N., Çetin, A., Cansız, A., Şekerci, M. & Dinçer, M. (2004). *Acta Cryst.* E**60**, o1580–o1582.

[bb4] Kumaran, D., Ponnuswamy, M. N., Jayanthi, G., Ramakrishnan, V. T., Chinnakali, K. & Fun, H.-K. (1999). *Acta Cryst.* C**55**, 581–582.

[bb5] Sheldrick, G. M. (2008). *Acta Cryst.* A**64**, 112–122. 10.1107/S0108767307043930 18156677

[bb6] Stoe & Cie (2005). *X-AREA*, *X-SHAPE* and *X-RED32* Stoe & Cie, Darmstadt, Germany.

[bb7] Zamani, K., Faghihi, K., Sangi, M. R. & Zolgharnein, J. (2003). *Turk. J. Chem*, **27**, 119–125.

